# Predicting the prognosis, immune response, and immunotherapy in head and neck squamous cell carcinoma using a novel risk model based on anoikis-related lncRNAs

**DOI:** 10.1186/s40001-023-01521-9

**Published:** 2023-11-28

**Authors:** Hongxia Deng, Zhengyu Wei, Juan Du, Zhisen Shen, Chongchang Zhou

**Affiliations:** 1 Department of Otolaryngology-Head and Neck Surgery, Ningbo Medical Center Lihuili Hospital, Ningbo University, NingboZhejiang, 315040 China; 2 Health Science Center, Ningbo University, Ningbo, 315211 Zhejiang China

**Keywords:** HNSCC, Anoikis, lncRNAs, Immune response

## Abstract

**Background:**

Head and neck squamous cell carcinoma (HNSCC) is an extremely heterogeneous and metastatic disease. Anoikis, which is a specific type of programmed apoptosis, is involved in tumor metastasis, tissue homeostasis, and development. Herein, we constructed an anoikis-related long non-coding RNA (lncRNA) signature to predict the prognosis, immune responses, and therapeutic effects in HNSCC patients.

**Methods:**

A total of 501 HNSCC samples were acquired from the TCGA database and randomly classified into the training and validation groups (1:1 ratio). Thereafter, the results derived from the training set were analyzed with the LASSO regression analysis, and a novel anoikis-related lncRNA risk model was constructed. Time-dependent ROC curves and Kaplan–Meier analysis were carried out to assess the diagnostic value and survival outcomes. A nomogram was utilized to predict the prognostic accuracy. Furthermore, we studied the tumor microenvironment, tumor mutation burden, enrichment pathways, and the response to chemotherapy and immunotherapy.

**Results:**

Seven anoikis-related lncRNAs (AC015878.1, CYTOR, EMSLR, LINC01503, LINC02084, RAB11B-AS1, Z97200.1) were screened to design a novel risk model, which was recognized as the independent prognostic factor for HNSCC patients. The findings implied that low-risk patients showed significantly longer OS, PFS, and DSS compared to those high-risk patients. The two groups that were classified using the risk model showed significant differences in their immune landscape. The risk model also predicted that low-risk HNSCC patients could attain a better response to immunotherapy, while high-risk patients would be more sensitive to gemcitabine, docetaxel, and cisplatin.

**Conclusions:**

We constructed a novel risk model that could be employed for effectively predicting patient prognosis with a good independent prognostic value for HNSCC patients. Furthermore, this model could be used for designing new immunotherapeutic and chemotherapeutic strategies, and it helps clinicians establish personalized and detailed strategies for HNSCC patients.

## Introduction

Head and neck squamous cell carcinoma (HNSCC) is a prevailing type of cancer that affects the head and neck region, originating from the mucosal epithelium, especially the nasopharynx, larynx, oropharynx, and hypopharynx [1]. One of the most predominant pathological types of head and neck cancer is squamous cell carcinoma, which accounts for 90% of cases, and affects people’s health and lives. With more than 600,000 new diagnoses and over 300,000 deaths each year, it is the sixth most common cancer worldwide [2]. With the development of medical technologies, HNSCC patients with early stage showed a better prognosis, as they benefited from the combined treatment strategies, including surgery, radiotherapy, and chemotherapy [3]. However, patients with recurrent or metastasis are usually incurable and show a median survival duration of only 10 months [1]. The introduction of immune checkpoint blockade (ICB) has significantly improved patient outcomes, but about 80% of patients showed an unsatisfactory response to therapy owing to individual variability and drug resistance [4, 5]. In addition, most HNSCC patients were diagnosed at an advanced stage, which could be attributed to the lack of effective early diagnosis, leading to a 5-year OS rate of less than 50% [6]. Hence, reliable predictive biomarkers must be identified for improving the prognosis of HNSCC patients.

Anoikis was first described in 1994, and is a specific type of programmed apoptosis process, which occurs due to the detachment of cells from the extracellular matrix (ECM) [7]. It is involved in tissue homeostasis, disease occurrence, and tumor metastasis [8]. It acts as a protective mechanism and helps in regulating the uncontrolled growth of dysplastic cells or ectopic somatic cells. However, during the infiltration and metastasis of malignant neoplasms, tumor cells exhibit anoikis resistance and are released from their cell–ECM and cell–cell adhesion states. After they are released, they survive, disseminate, and metastasize in the circulatory system by resisting the anoikis-induced tumor cell death [9, 10]. Recurrence and metastasis are issues that disturb the patients and doctors. Hence, further research in anoikis is necessary to effectively optimize human cancer therapeutic strategies.

In the past, long non-coding RNAs (lncRNAs) have garnered a lot of scientific interest owing to their important role in cancer progression, including proliferation, migration, metastasis, immune evasion, and tumor prognosis [11]. lncRNAs are described as noncoding transcripts with > 200 nucleotides [12]. To date, lncRNAs were seen to be closely related to anoikis resistance in several tumors. In breast cancer, APOC1P1-3 inhibited early apoptosis of cancer cells and enhanced anoikis resistance by decreasing the activated poly ADP-ribose polymerase (PARP) and Caspase 3, 8, 9 levels [13]. In lung adenocarcinoma, LINC01546 acts as a pro-metastatic molecule and is necessary for AKT-induced tumor infiltration, metastasis, and anoikis resistance [14]. MRPL23-AS1 increased the tumor cell anoikis resistance in salivary adenoid cystic carcinoma by using the zeste homolog 2 (EZH2) enhancer at the p19INK4D promoter region [15]. Similarly, HOTAIR also regulates anoikis resistance by employing EZH2 and affecting H3K27 methylation in ovarian cancer cells [16]. Nevertheless, none of the researchers have explored the involvement of anoikis-related lncRNAs in HNSCC patients to date.

The relationship between lncRNAs and anoikis served as the basis for constructing the prognostic scoring model based on the anoikis-related lncRNAs in HNSCC. In addition, we assessed the relationship between the risk model and tumor somatic mutation burden (TMB) and the immunological features and established its clinical predictive value for predicting the efficacy in terms of immunotherapy and chemotherapy response.

## Methods

### Data collection

**Fig. 1:**
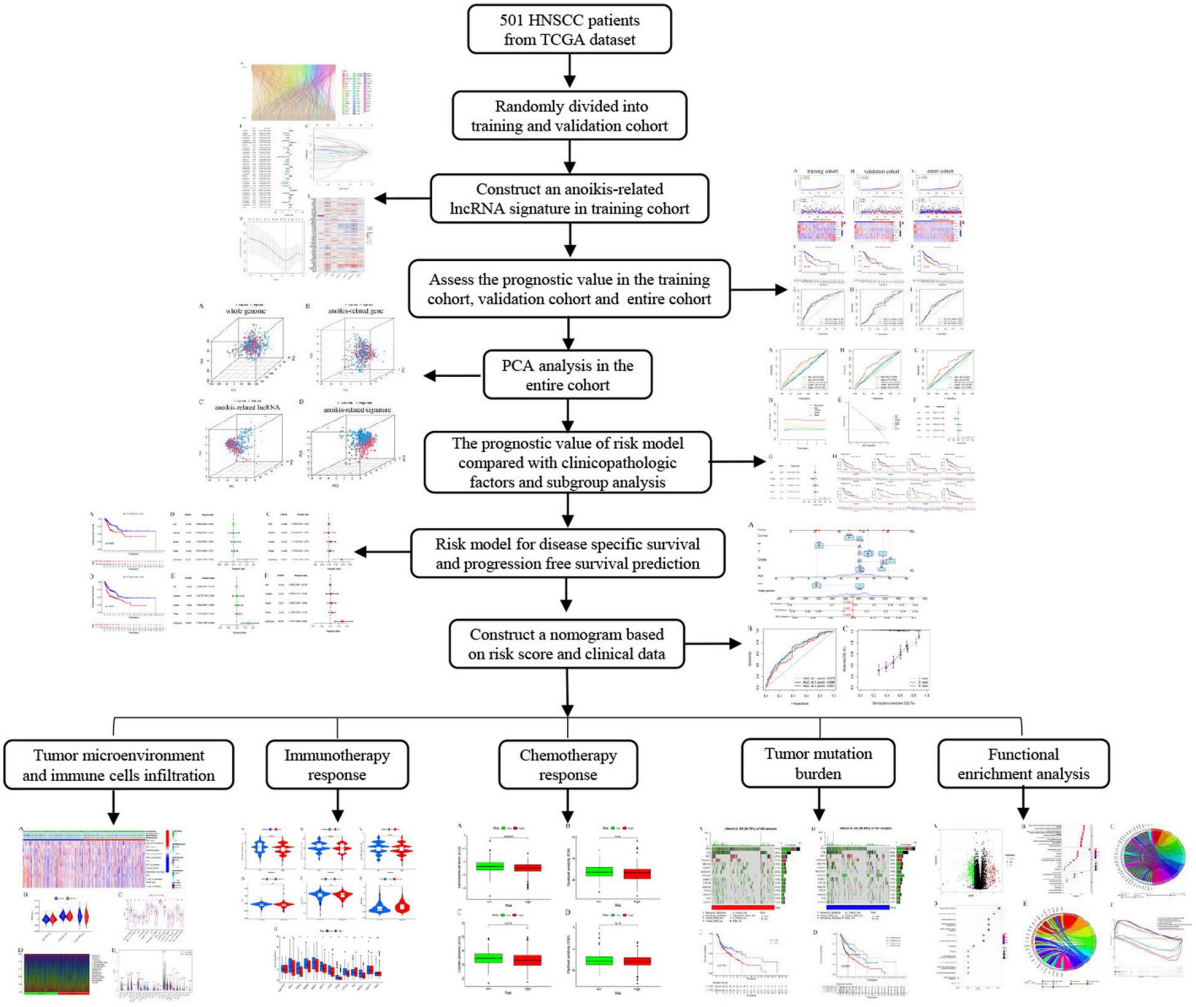
Flowchart of the strategy used in this study

We downloaded all data for TCGA–HNSC project from The Cancer Genome Atlas database (TCGA, https://portal.gdc.cancer.gov/, updated February 12, 2023). 501 HNSCC samples with complete prognostic data were filtered out. Then, the data comprised transcriptomic profiling information and corresponding clinicopathological were downloaded. Table 1 depicts the detailed clinical features of HNSCC patients. The 501 HNSCC samples were categorized into training and validation cohorts (sets, 1:1 ratio) for constructing and validating the risk model. Figure 1 presents the flowchart implemented in this study. It is important to note that TCGA is a publicly accessible and open-access database, hence, no patient consent for participation or institutional ethical approval was necessary.

**Table 1:** Clinical features of the HNSCC patients included in this study

Covariates	Type	Entire		Training		Validation		*P* value
		Number	Percent	Number	Percent	Number	Percent	
Age								
	≤ 60	246	49.10	116	46.40	130	51.79	0.2636
	> 60	255	50.90	134	53.60	121	48.21	
Gender								
	Female	133	26.55	70	28	63	25.10	0.5261
	Male	368	73.45	180	72	188	74.90	
Smoking history								
	No	113	22.55	58	23.20	55	21.91	0.9418
	Yes	378	75.45	187	74.80	191	76.10	
	Unknown	10	2	5	2	5	1.99	
Alcohol history								
	No	158	31.54	82	32.80	76	30.28	0.285
	Yes	332	66.27	165	66	167	66.53	
	Unknown	11	2.20	3	1.20	8	3.19	
Histopathological grade								
	G1	61	12.18	31	12.40	30	11.95	0.5758
	G2	299	59.68	150	60	149	59.36	
	G3	119	23.75	61	24.40	58	23.11	
	G4	2	0.40	–	–	2	0.80	
	Unknown	20	3.99	8	3.20	12	4.78	
T classification								
	T1	33	6.59	16	6.40	17	6.77	0.0649
	T2	144	28.74	59	23.60	85	33.86	
	T3	130	25.95	75	30	55	21.91	
	T4	179	35.73	94	37.60	85	33.86	
	Unknown	15	2.99	6	2.40	9	3.59	
N classification								
	N0	239	47.70	113	45.20	126	50.20	0.6737
	N1	80	15.97	45	18	35	13.94	
	N2	153	30.54	78	31.20	75	29.88	
	N3	7	1.40	4	1.60	3	1.20	
	Unknown	22	4.39	10	4	12	4.78	
Metastasis status								
	M +	25	4.99	15	6	10	3.98	0.535
	M0	471	94.01	233	93.20	238	94.82	
	Unknown	5	1	2	0.80	3	1.20	
Stage								
	Stage I	19	3.79	9	3.60	10	3.98	0.0938
	Stage II	95	18.96	36	14.40	59	23.51	
	Stage III	102	20.36	57	22.80	45	17.93	
	Stage IV	271	54.09	142	56.80	129	51.39	
	Unknown	14	2.79	6	2.40	8	3.19

### Identifying the anoikis-related lncRNAs

We also acquired 65 anoikis-related genes from the GeneCards database (https://www.genecards.org) with relevance scores > 2. The gene expression levels were extracted with the “limma” package from the training cohorts. We then conducted Pearson correlation analysis for screening the co-expressed lncRNAs using the “igraph” and “reshape2” packages, with the threshold set at *p* < 0.001 and |Pearson *R*|> 0.4. We also conducted the univariate regression analysis to identify the anoikis-related lncRNAs based on their prognostic value (*p* values < 0.05).

### Constructing the prognostic risk model

Here, we employed the Least Absolute Shrinkage and Selection Operator (LASSO) regression analysis to identify the anoikis-related lncRNAs related to the survival data-based prognosis with the ‘glmnet’ tool in R software. Finally, 7 lncRNAs (AC015878.1, CYTOR, EMSLR, LINC01503, LINC02084, RAB11B-AS1, Z97200.1) were shortlisted for designing the prognostic signature. The below-mentioned formula was used for computing the risk score for every HNSCC sample based on the coefficients by means of multivariate regression analysis. The medium risk score was designated as a baseline value for classifying the training, validation, and entire cohorts using the data derived from both high- and low-risk groups:

$$ {\text{Risk}}\;{\text{score}} = \sum\nolimits_{(i = 1)}^{n} {{\text{coefi}} \times {\text{anoikis - related}}\;{\text{lncRNA}}\;{\text{expression}}} $$

### Comprehensive assessment of prognostic value for the risk model

The scatter plots and risk curves were used to depict the distribution and survival status regarding the risk scores for all HNSCC patients. The difference in the overall survival (OS) rate between both risk groups was presented with the Kaplan–Meier (K–M) curves generated by the log-rank test. We generated the time-dependent receiver operator characteristic (ROC) curves with the risk model for determining the 1-, 3-, and 5-year OS rates with the help of the “timeROC” and “survivalROC” R packages using the data derived from the training, validation, and entire cohorts respectively. To comprehensively ascertain the efficacy of the risk model, the entire cohorts were employed for subsequent evaluation. The “Rtsne” and “ggplot2” packages were used to carry out Principal component analysis (PCA) and assess if the anoikis-related lncRNA signature, whole genome, anoikis-associated genes, and anoikis-associated lncRNAs could categorize the HNSCC patients into the high- and low-risk groups. Prognostic efficacy was compared between age, gender, grade, stage, and risk model using multivariate ROC curves, C-index analysis, and decision curves. Thereafter, the univariate and multivariate regression analyses were implemented to assess the independent prognosis-predictive value of the risk model. Subgroup analysis of OS was undertaken to study the implementation of the probable risk model depending on the gender, histopathological grade, age, and clinical stage. Furthermore, the log-rank test was utilized for comparing the progression-free survival (PFS) and the disease-specific survival (DSS) values in both risk groups, while the prognostic significance of DSS and PFS was also evaluated using the Cox regression analyses. In addition, the clinical data and risk scores were used to develop the nomogram to anticipate survival probability. We employed the time-dependent ROC curves and calibration plots for determining the performance of the proposed nomogram to anticipate the survival of patients.

### Tumor microenvironment (TME) and the infiltration levels of the immune cells

We calculated the immune-, stromal-, and ESTIMATE scores of TME for both risk groups using the ESTIMATE algorithm and plotted the heatmap and violin plots. Furthermore, the enrichment scores for the 13 immune-linked pathways were compared for each HNSCC specimen using the single sample gene-set enrichment analysis (ssGSEA) process. The relative proportion of 22 tumor-infiltrating immune cells in both risk groups was computed using the CIBERSORT analysis.

### Immunotherapy and chemotherapy

Here, we have also studied whether the risk scores could serve as biomarkers for depicting the clinical responses of patients to chemotherapy and immunotherapy. For this purpose, the immunophenoscores (IPSs) were acquired from the TCGA–HNSC project of The Cancer Immunome Atlas (TCIA) database (https://tcia.at/home). Thereafter, IPS values in both groups were compared. Finally, the expression levels of 13 ICB genes (HAVCR2, IDO1, CD8A, GZMB, GZMA, PRF1, LAG3, IFNG, CTLA4, TNF, PDCD1, CD274, and TBX2) were compared between the two risk groups. In addition, four clinically used common chemotherapeutic drugs (like docetaxel, gemcitabine, cisplatin, and paclitaxel) were considered to assess the chemotherapy response by comparing the half-maximum inhibitory concentration (IC_50_), which was computed with the “pRRophetic” software.

### TMB analysis

The somatic mutations determined in both risk groups were calculated and visualized by the “maftool” and “GenVisR” packages. The TMB score was generated by dividing the sum of somatic mutations by the exome size [17]. Log-rank tests and K–M curves were determined to assess the OS values in high- and low-TMB groups by utilizing the “survival” and “survminer” packages.

### Functional annotation of the risk model

The differentially expressed genes (DEGs) between both risk groups were identified with the thresholds set as log_2_-fold change (|log_2_FC|) > 1 and false discovery rate (FDR)* p* < 0.05, using “DESeq2” software. The DEGs results were used to carry out Kyoto Encyclopedia of Genes and Genomes (KEGG) and the Gene ontology (GO) enrichment analyses to investigate the probable signaling pathways and biological functions, with the help of the “clusterProfiler” and “ggplot2” tools. In addition, the Gene Set Enrichment Analysis (GSEA) was employed to determine the activated functional pathways in both risk groups that fulfilled the FDR *p* < 0.05 criterion.

### Statistical analysis

R software (ver. 4.1.0) was employed for analyzing the data and plotting the graphs. Wilcoxon and Chi-squared tests were used for analyzing the continuous and categorical variables, respectively. Data with *p* values < 0.05 were termed statistically significant.

## Results

### Identifying the prognostic anoikis-related lncRNAs in HNSCC tissues

**Fig. 2:**
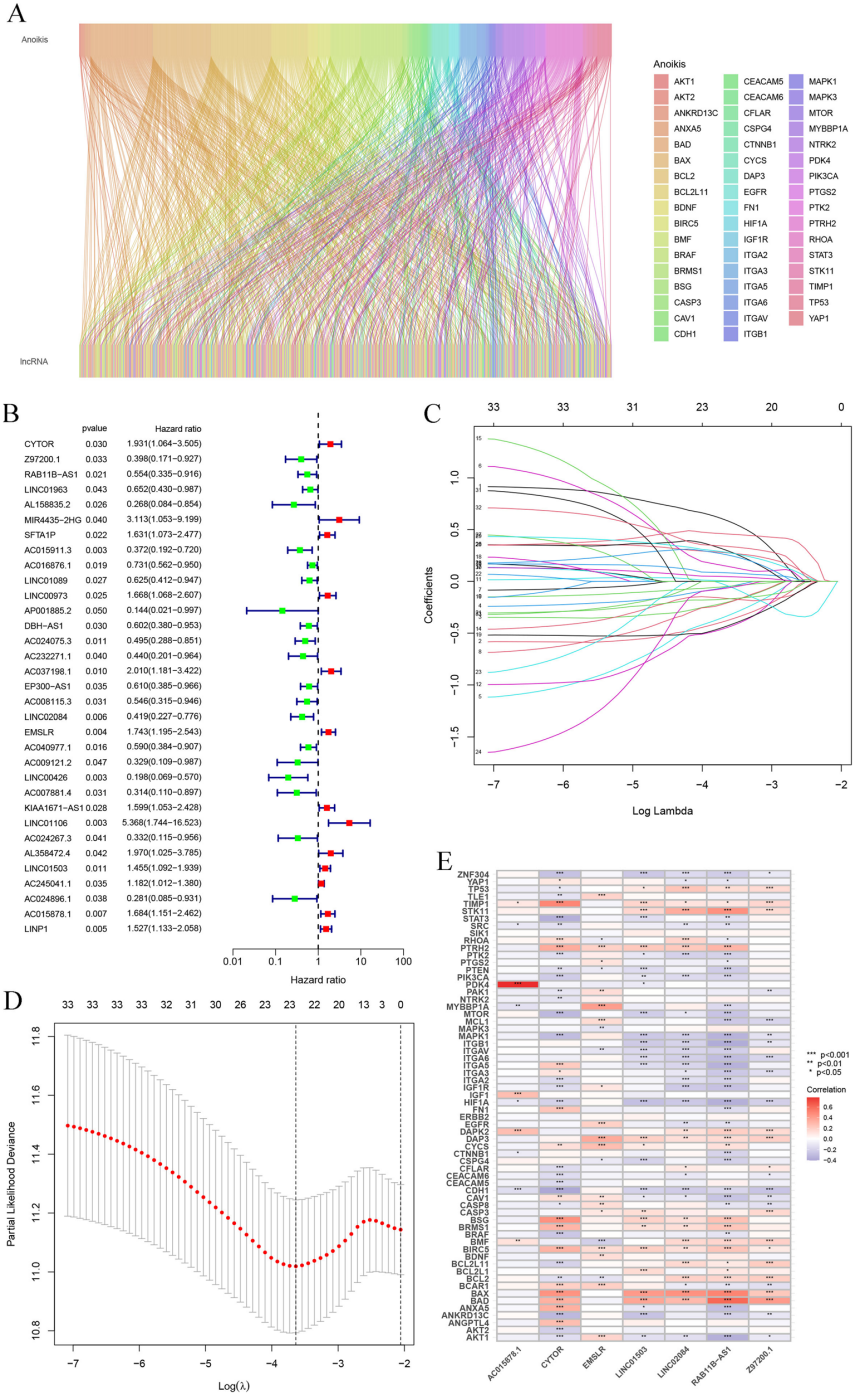
Constructing the anoikis-related lncRNA signature in the training set. **A** Sankey diagram depicting the relationship between anoikis-related genes and co-expressed lncRNAs; **B** forest plot of 33 prognostic anoikis-related lncRNAs using the univariate regression analysis (*p* < 0.05); **C** distribution of the LASSO coefficients of the selected anoikis-related lncRNAs; **D** generalized cross-validation curve of the optimal parameter (*λ*) selection based on the minimum criteria; **E** relationship between the seven prognostic lncRNAs and anoikis-related genes

A total of 533 anoikis-related lncRNAs were filtered by Pearson correlation analysis using the 65 anoikis-related gene expression levels determined for 501 HNSCC patients from the TCGA database. These data have been presented using the Sankey diagram (Fig. 2A). In addition, we also screened 33 prognostic lncRNAs using the univariate regression analysis, and assessed the prognostic anoikis-related lncRNAs in this study (*p* < 0.05) (Fig. 2B). The 501 HNSCC samples were classified into the training and validation sets (ratio of 1:1). In addition, the variables included in the training set were decreased with the LASSO Cox regression analysis technique by introducing the lambda value (Fig. 2C, D). Finally, seven lncRNAs (AC015878.1, CYTOR, EMSLR, LINC01503, LINC02084, RAB11B-AS1, Z97200.1) were detected for constructing the prognostic risk model. Here, every sample was allotted a risk score using the corresponding coefficients (Table 2). The association between the seven lncRNAs and anoikis-related genes is shown in Fig. 2E.

**Table 2:** Coefficients of seven anoikis-related lncRNAs used in the risk model

LncRNA	Coefficient
CYTOR	1.076602309
Z97200.1	− 1.047843038
RAB11B-AS1	− 0.725763181
LINC02084	− 0.837969986
EMSLR	0.5142609
LINC01503	0.462273641
AC015878.1	0.8283574

### Assessing the risk model integrating seven anoikis-related lncRNAs

**Fig. 3:**
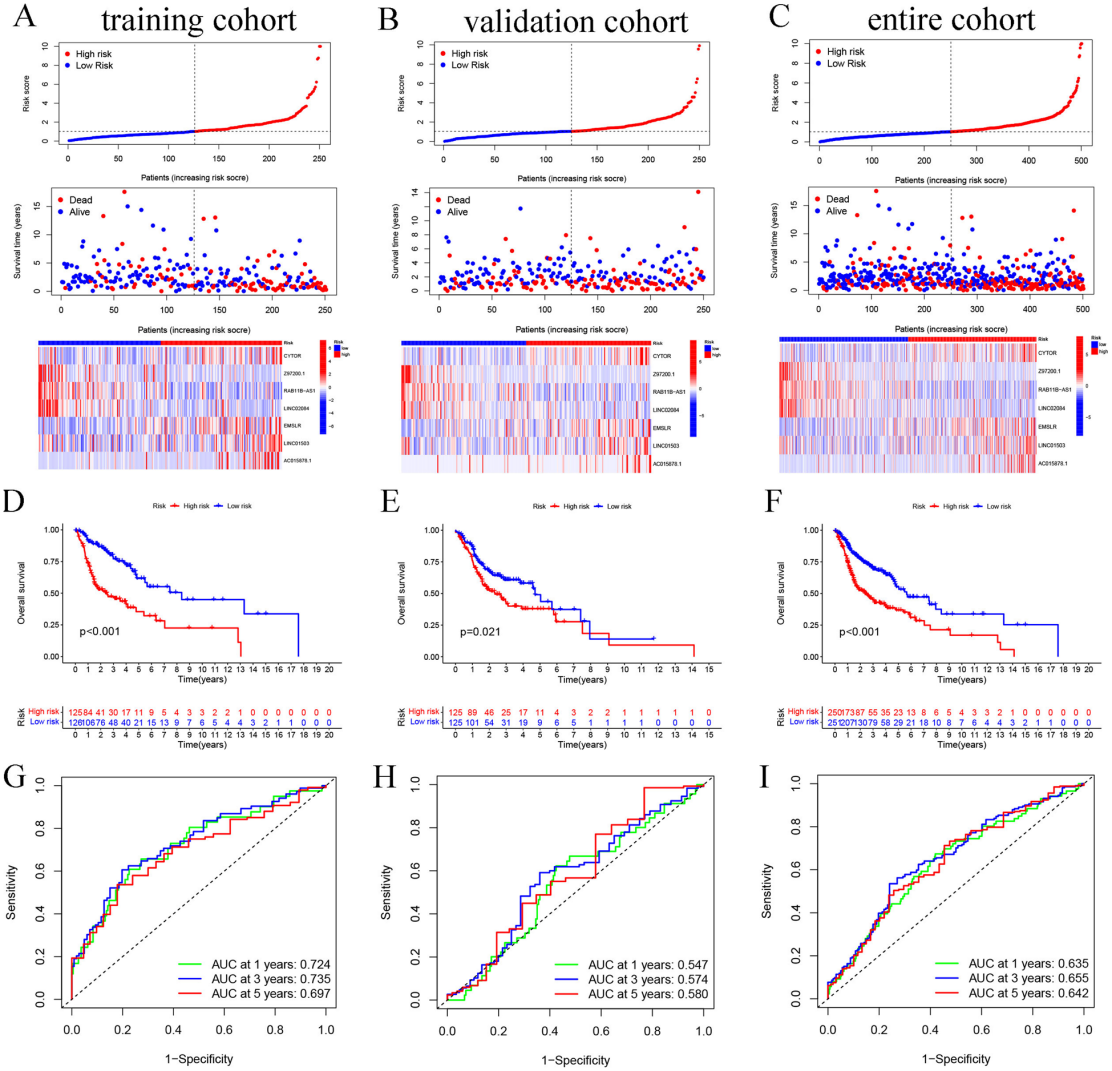
Assessment of prognostic values in the training, validation, and entire data sets. Comparison of the OS status of HNSCC patients with varying risk scores, and heatmap depicting the anoikis-related lncRNA signature in **A** training, **B** validation, and **C** entire data sets. K–M curves showed the OS of both the risk HNSCC groups in **D** training (log-rank, *p* < 0.001), **E** validation (log-rank, *p* = 0.021), and **F** entire sets (log-rank, *p* < 0.001). The AUC values for the time-dependent ROC curves depict the OS prediction values for **G** training, **H** validation, and **I** entire cohorts

HNSCC patients were categorized into two risk groups with the help of a median risk score that was computed using the anoikis-related lncRNA model. We plotted the risk curves and scatter plots to show the distribution and OS rates of HNSCC patients included in the training, validation, and entire data sets, respectively. The heatmap implied that the high-risk HNSCC patients in the different data sets showed an upregulation in the CYTOR, EMSLR, LINC01503, and AC015878.1 expression levels (Figs. 3A–C). The findings of the K–M curves implied that the low-risk patients showed a longer OS duration than the high-risk patients in the training set (Fig. 3D, p < 0.001), validation set (Fig. 3E, p = 0.021), and entire data set (Fig. 3F, p < 0.001). Time-dependent ROC analysis showed the AUC values of the risk score in the training cohort, validation cohort, and entire cohort, respectively (Fig. 3G–I).

### Determining the independent prognosis-predictive value of the risk model

**Fig. 4:**
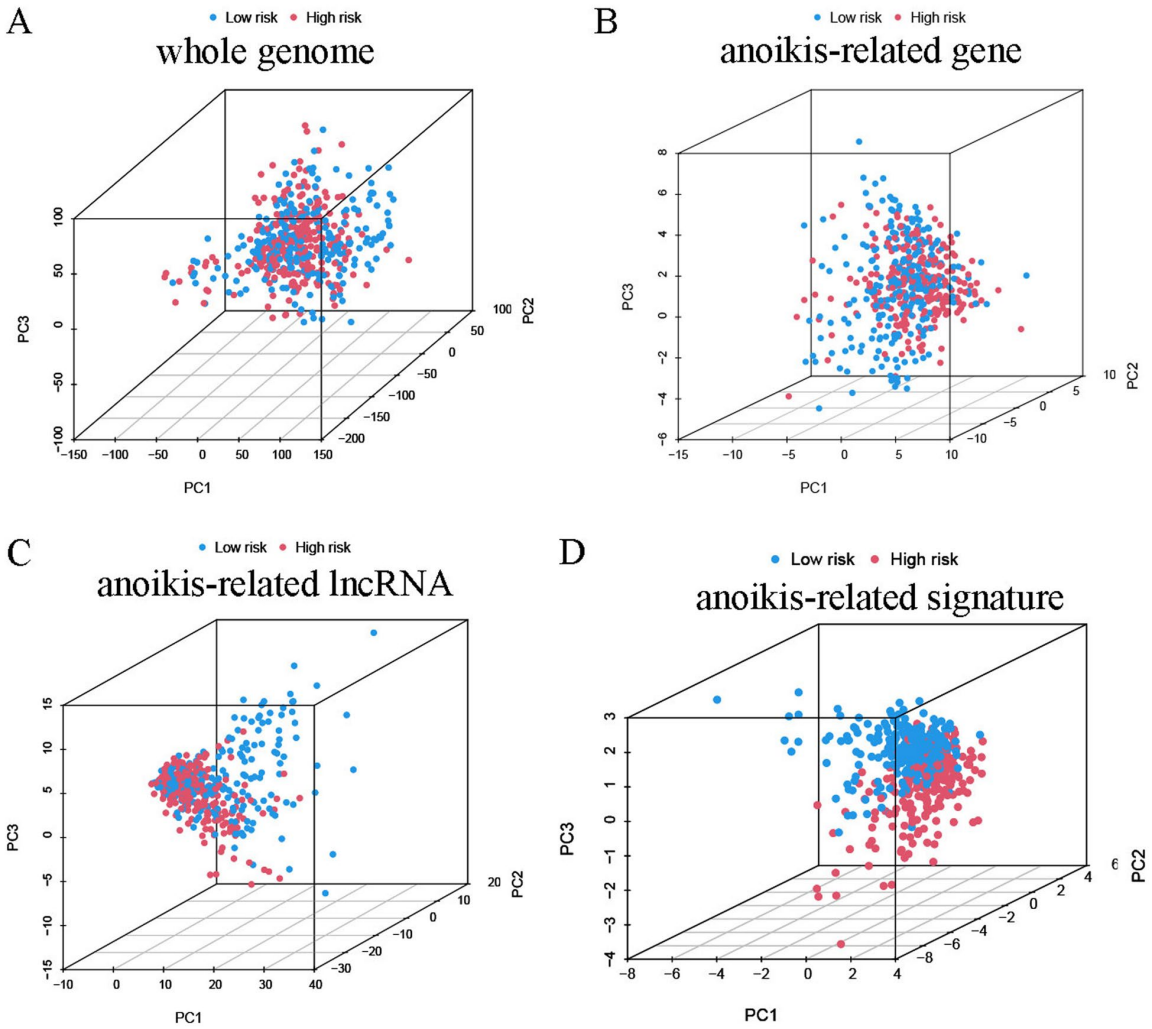
PCA analysis of the data derived from the entire cohort. The results of the PCA analyses indicated that the **D** anoikis-related lncRNA signature could be differentiated based on the risk status of HNSCC patients compared to the **A** whole genome, **B** anoikis-related gene and **C** anoikis-related lncRNA

**Fig. 5:**
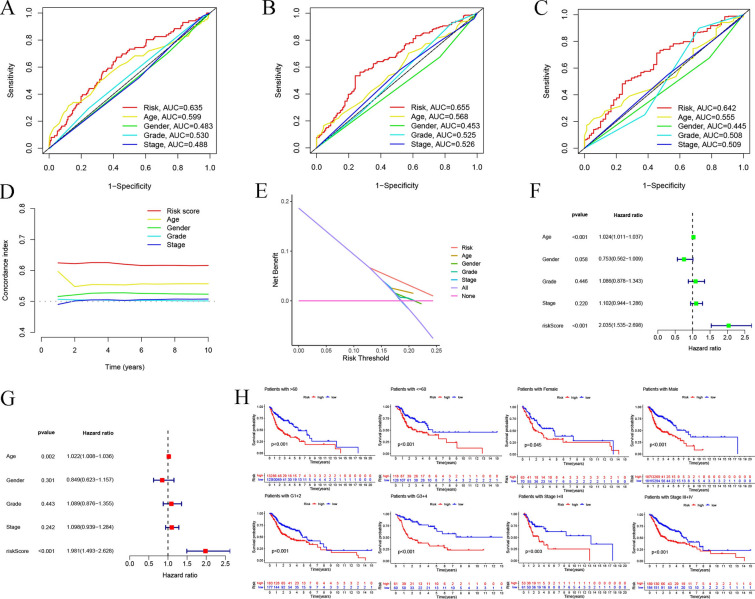
Predictive value depicted by the proposed risk model in comparison to clinicopathologic factors and subgroup analysis. The findings of the ROC curves of prognostic accuracy were used for comparing the risk score with clinicopathologic factors in the entire set for **A** 1 year, **B** 3 years, and **C** 5 year. **D** C-index was employed for comparing the prognostic accuracy of clinical factors and risk score. **E** Decision curves depicted the clinical advantages described by the risk model using the entire cohort. **F** Univariate and **G** multivariate regression analysis of risk scores in the entire set. **H** Subgroup analysis of the K–M survival curve depends on factors like gender, age, histopathological grade, and clinical stage

**Fig. 6:**
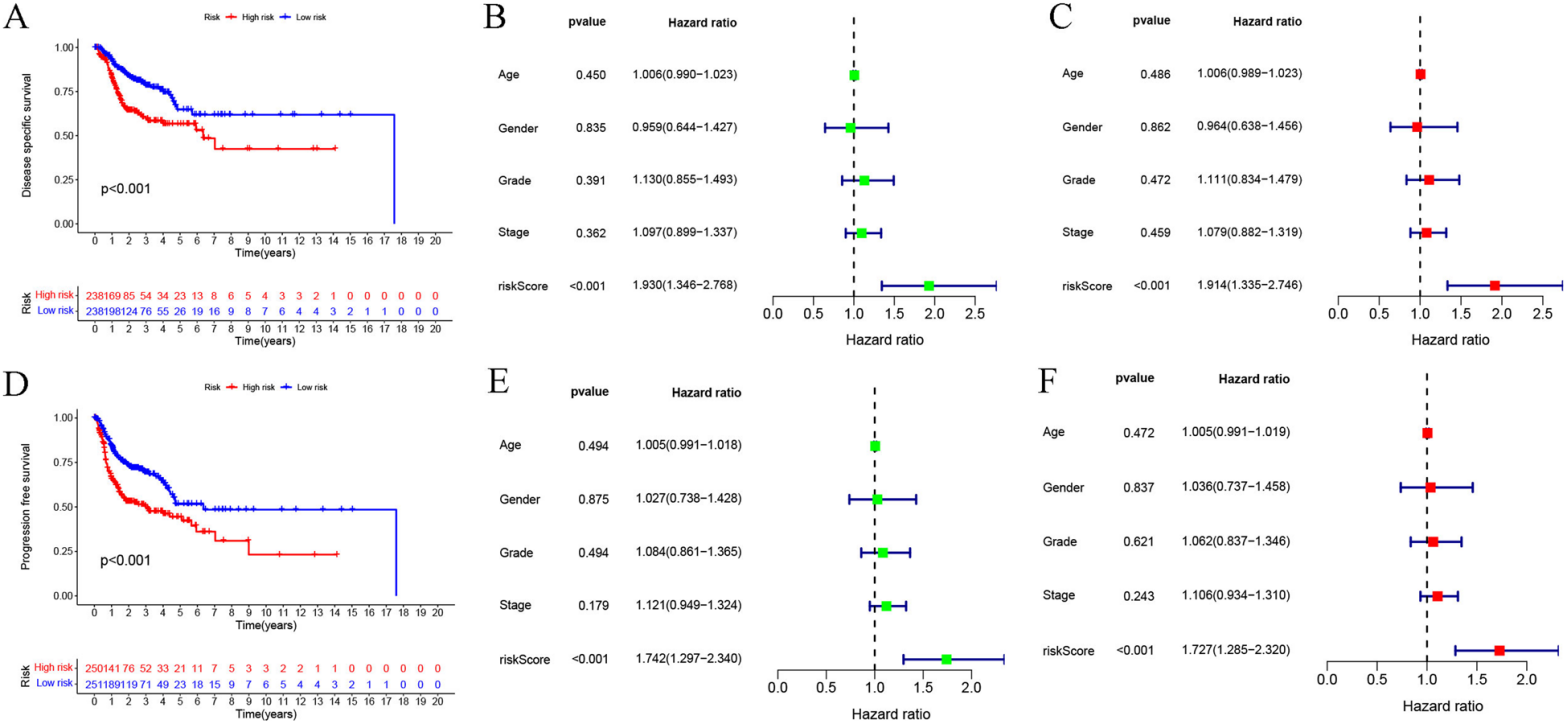
Risk model for DSS and PFS prediction. **A** K–M curves depicted the DSS values in both risk groups (log-rank, *p* < 0.001). **B** Univariate and **C** multivariate regression analyses of DSS for the proposed risk model. **D** K–M curves depicted the PFS of both risk groups (log-rank, *p* < 0.001). **E** Univariate and **F** multivariate regression analysis of PFS for the risk model

**Fig. 7:**
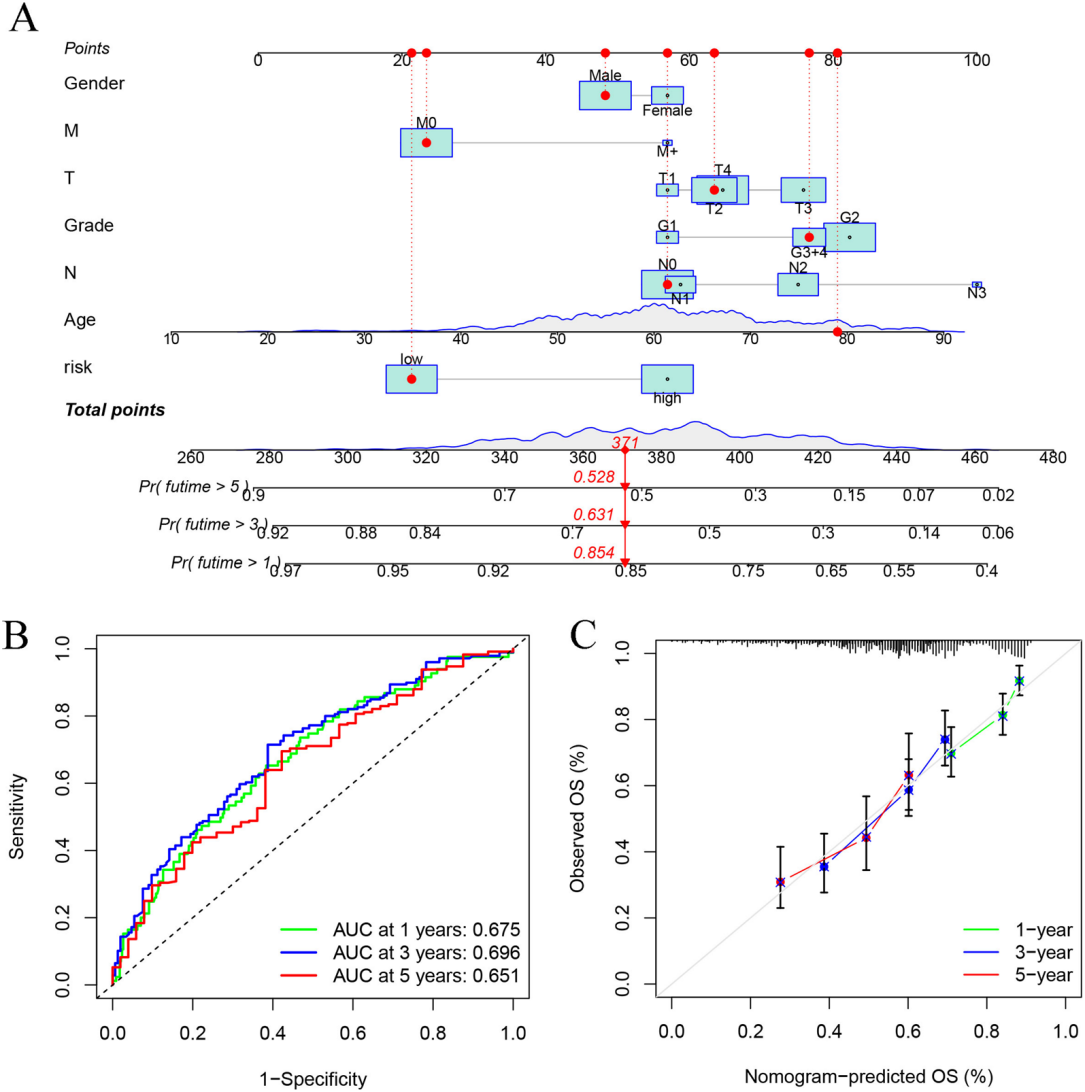
Constructing a nomogram depending on the risk scores and clinical data. **A** Signature-based nomogram was used to predict the probability of 1-, 3- and 5-year OS. **B** AUC values of time-dependent ROC curves for predicting the OS values. **C** Calibration plots of the nomogram to anticipate the 1-, 3-, and 5-year OS

The probable predictive value of anoikis-related lncRNA risk model had been preliminarily assessed using the training and validation sets, however, the entire data set was employed for the comprehensive and accurate analysis. PCA analysis was employed for visualizing the distribution of HNSCC patients, and the findings indicated that the anoikis-related lncRNA signature could help in differentiating the HNSCC patients depending on their risk score values (Fig. 4). The ROC curves at 1, 3, and 5 years indicated that the risk model exhibited better results compared to those displayed by other clinical prognostic indicators, like gender, age, grade, and clinical stage (Fig. 5A–C). C-index analysis implied that the risk score exhibited better prognostic accuracy compared to that displayed by other clinical factors (Fig. 5D). Decision curve analysis implied that the risk model presented the optimal clinical benefit in comparison to gender, age, grade, and clinical stage (Fig. 5E). In addition, Cox regression analyses were conducted and it was seen that the proposed risk model can be employed as the independent predictive factor for HNSCC [univariate HR = 2.035, 95%CI = 1.535–2.698 (Fig. 5F); multivariate HR = 1.981, 95%CI = 1.493–2.628 (Fig. 5G); *p* < 0.001]. K–M analysis of clinical subgroup characteristics indicated that low-risk HNSCC patients exhibited a significantly better OS value compared to high-risk patients (Fig. 5H; *p* < 0.05). Moreover, low-risk HNSCC patients displayed longer DSS (*p* < 0.001, Fig. 6A) and PFS (*p* < 0.001, Fig. 6D) durations in comparison to those presented by high-risk patients. Further analyses implied that the proposed risk model can serve as an independent prognosis-predictive factor for DSS and PFS (univariate, Fig. 6B, E; multivariate, Fig. 6C, F; *p* < 0.001). We developed a nomogram depending on the risk scores and clinical parameters like age, metastasis, gender, T stage, grade, and N stage, for predicting the 1-, 3-, and 5-year OS rates of HNSCC patients (Fig. 7A). Time-dependent ROC curves revealed that the proposed nomogram presented a good survival prediction using AUC values > 0.5 (1 year: 0.675; 3 years: 0.696; 5 years: 0.651) (Fig. 7B). Furthermore, the calibration curves revealed that the predicted line was next to the 1-, 3-, and 5-year ideal lines (ideal curve) (Fig. 7C).

### Correlation of the TME, infiltration levels of immune cells, and risk scores

**Fig. 8:**
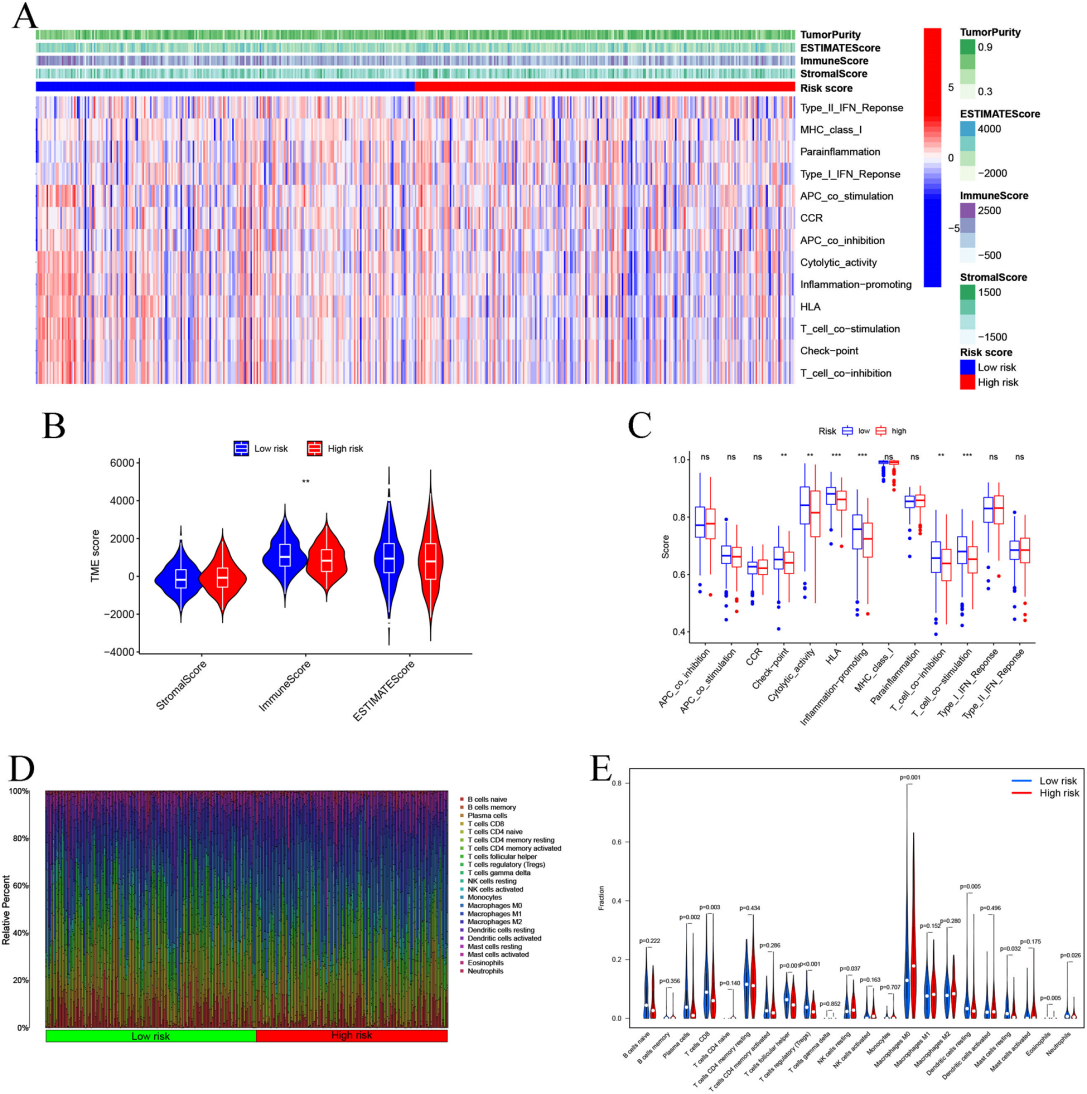
Relationship between the immune cells infiltration, TME, and anoikis-related lncRNA signature. **A** Heatmap shows the immune score, ESTIMATE score, stromal score, tumor purity, and immune-associated pathway in both risk groups. **B** Comparison of TME scores in both the risk groups using the ESTIMATE algorithm. **C** Box plot presents the comparison of 13 immune-linked functions in both risk groups. **D** Distribution of 22 tumor-infiltrating immune cells in all HNSCC patients quantified using the CIBERSORT algorithm. **E** Violin plot shows the fraction of 22 immune cells in both risk groups. *p < 0.05, **p < 0.01, ***p < 0.001, *ns* not significant

Here, we employed the ESTIMATE algorithm for assessing the link between the TME status and risk score among HNSCC patients. The findings implied that low-risk patients showed significantly higher immune scores than high-risk patients (Fig. 8A, B). This phenotype implied that the risk scores might be reversely linked to the immune status. Here, we conducted the ssGSEA analysis and the resulting findings implied that the low-risk patients showed a higher enrichment in their checkpoint, inflammation-promoting, human leukocyte antigen (HLA), T-cell co-inhibition, cytolytic activity, and T-cell co-stimulation pathways (Fig. 8A, C). We also employed the CIBERSORT algorithm to quantify the relative proportion of 22 tumor-infiltrating immune cells related to risk scores in every HNSCC patient (Fig. 8D). It was observed that low-risk patients had a high infiltration level of CD8 T cells, follicular helper T cells, plasma cells, resting dendritic cells, resting mast cells, and regulatory T cells than high-risk patients (Fig. 8E).

### Impact of the risk model on patient response to chemotherapy and immunotherapy

**Fig. 9:**
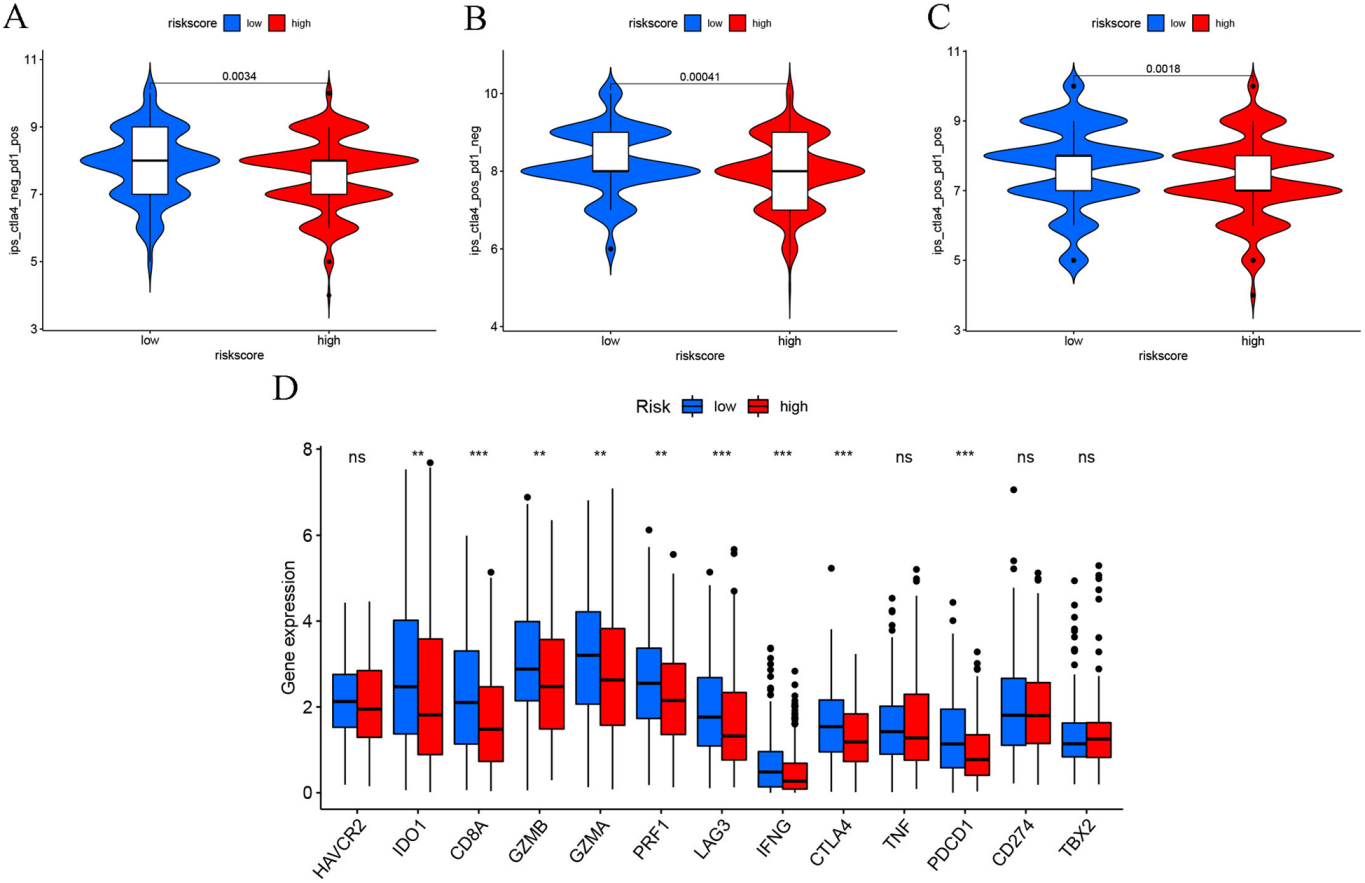
Impact of anoikis-related lncRNA signature on immunotherapy response. Correlation of the anoikis-related lncRNA signature and IPS for **A** anti-PD1 immunotherapy, **B** anti CTLA4 monotherapy and **C** combined anti-PD1 with anti-CTLA4 immunotherapy. **D** Comparison of the immune-linked gene expression levels between the two risk groups

**Fig. 10:**
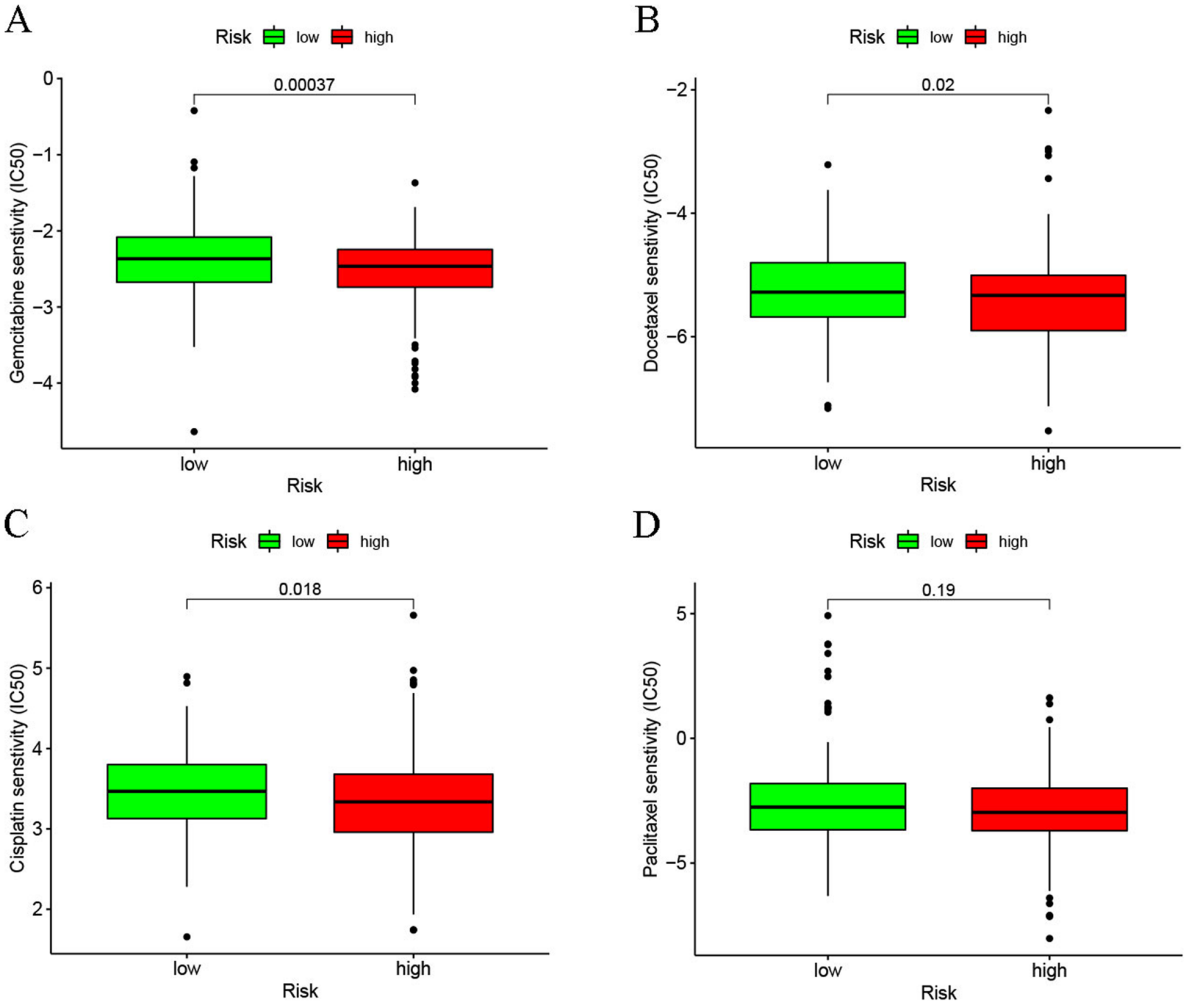
Effect of anoikis-related lncRNA signature on chemotherapy response. High-risk HNSCC patients showed significantly low IC_50_ values for **A** gemcitabine, **B** docetaxel, **C** cisplatin; and **D** paclitaxel

The violin plots that were presented using the IPS values denoted that low-risk patients exhibited a good response to the treatment strategy using either a programmed cell death-1 (PD-1) inhibitor (*p* = 0.0034, Fig. 9A) or a cytotoxic T-lymphocyte associated protein 4 (CTLA4) inhibitor alone (*p* = 0.0018, Fig. 9B) and the combination of PD-1 and CTLA4 inhibitors (*p* = 0.0018, Fig. 9C). Thus, it could be concluded that low-risk patients exhibited a good response to immune checkpoint inhibitors. Furthermore, we compared the gene expression levels of 13 common ICB genes between both risk groups. The findings indicated that except for HAVCR2, TNF, CD274, and TBX2 genes, expression levels of the remaining 9 immune-associated genes (IDO1, CD8A, GZMB, GZMA, PRF1, LAG3, IFNG, CTLA4, and PDCD1) were significantly elevated in the low-risk patients (Fig. 9G) than the higher risk group. Furthermore, we derived the IC_50_ of each HNSCC patient using the pRRophetic algorithm to identify the link between the chemotherapy response and risk scores. The high-risk patients displayed significantly low IC_50_ values for gemcitabine (*p* < 0.001, Fig. 10A), docetaxel (*p* = 0.02, Fig. 10B), and cisplatin (*p* = 0.018, Fig. 10C), while patients in both groups showed no significant difference for paclitaxel (*p* = 0.19, Fig. 10D). These results implied that high-risk patients showed a higher sensitivity to gemcitabine, docetaxel, and cisplatin drugs.

### Correlation between the risk model and TMB in the HNSCC patients

**Fig. 11:**
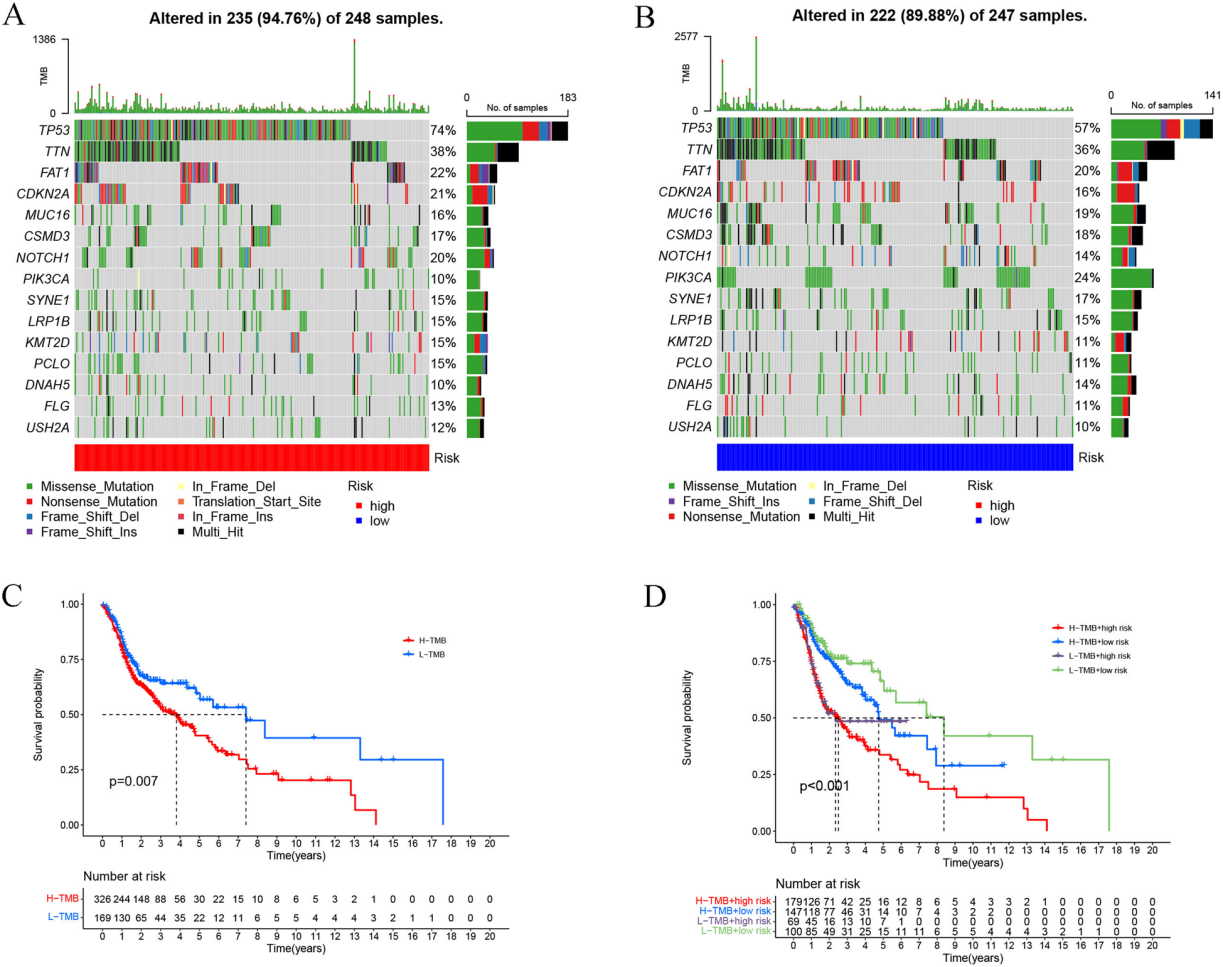
Relationship between the proposed risk model and TMB in HNSCC patients. Waterfall chart depicts the 15 top mutation genes in **A** high-risk and **B** low-risk patients. **C** K–M curves presented the OS values in both the TMB groups (log-rank, *p* = 0.007). **D** OS values of the HNSCC patients that were categorized as per the TMB states and risk scores (log-rank, *p* < 0.001)

To assess whether somatic mutations are relevant to the risk model, we compared the mutation frequencies of the genomic genes in the two groups. The high-risk patients harbored more somatic mutations compared to low-risk patients (94.76% *vs.* 89.88%). The waterfall plots displayed the top fifteen mutated genes in both risk groups, and the findings revealed that low-risk HNSCC patients displayed a lower propensity for *TP53* mutations than high-risk patients (57% *vs.* 74%, respectively) (Fig. 11A, B). Here, we sorted the HNSCC patients into the high- and low-TMB categories depending on TMB values, for assessing the influence of TMB on OS rates of HNSCC patients. The data displayed by both groups were used for carrying out log-rank tests. The findings of the K–M curves indicated that higher TMB patients showed a worse prognosis (*p* = 0.007, Fig. 11C). In addition, the results of combined TMB and risk scores analyses also indicated that the HNSCC patients with low TMB scores displayed better OS duration compared to low- or high-risk patients (*p* < 0.001, Fig. 11D).

### Functional analysis of the anoikis-related lncRNA risk model

**Fig. 12:**
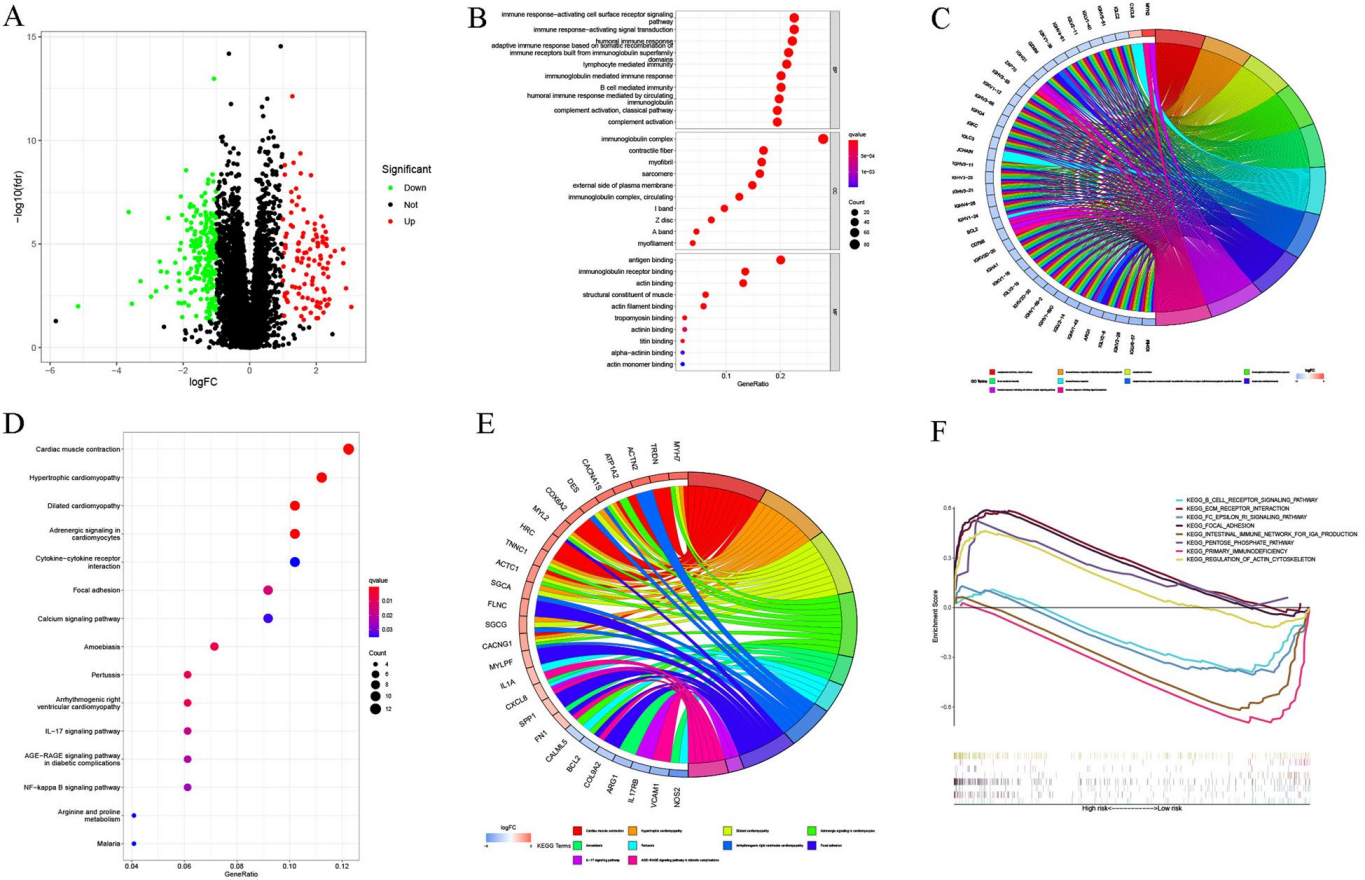
Functional analysis of anoikis-related lncRNA signature. **A** DEGs determined in both risk groups were presented using Volcano plots. The biological function of GO enrichment in both risk categories was depicted employing the **B** bubble chart and **C** circle plot. The biological pathways describing the KEGG analysis in both groups were depicted using the **D** bubble chart and **E** circle plot. **F** GSEA analysis was employed to compare the biological pathways between both risk groups

Here, we also identified the DEGs between the two risk groups and plotted them on a volcano diagram (Fig. 12A). GO analysis of the DEGs showed significant enrichment of many immune-associated biological processes, such as B-cell-mediated immunity, lymphocyte-mediated immunity, immune response, by circulating immunoglobulin, mediated humoral immune response, and complement activation classical pathways (Fig. 12B). The chord diagram further confirmed that DEGs were more enriched in immune-associated GO terms (Fig. 12C). KEGG pathway analysis suggested that the above DEGs were significantly enriched in the cardiomyocyte-associated pathways (Fig. 12D, E). The findings of GSEA enrichment analysis implied that the low-risk patients displayed a significant enrichment of immune-associated pathways, like intestinal immune network for IGA production, Fc epsilon RI signaling pathway, B cell receptor signaling pathway, and primary immunodeficiency. However, the high-risk patients were significantly enriched in cancer-associated pathways like the pentose phosphate pathway, focal adhesion, ECM receptor interaction, and the control of the actin cytoskeleton (Fig. 12F).

## Discussion

Anoikis is an important programmed cell death process that prevents the re-adhesion and growth of the shed cells or attachment of the shed cells to an incorrect location during the body’s development [18]. However, in many cancers, anoikis resistance is recognized as a primary mechanism for tumor invasion and migration, metastasis, and treatment resistance [19–21]. HNSCC is an insidious onset and highly invasive neoplasm, wherein a majority of the patients were diagnosed with metastatic carcinoma. It showed a low 5-year OS rate because of the lack of an effective early diagnosis and drug resistance strategy [1]. Thus, the construction of anoikis-related predictive models may help in effectively guiding the prognosis and treatment of HNSCC patients.

Herein, we construct a risk model consisting of seven anoikis-related lncRNAs (AC015878.1, CYTOR, EMSLR, LINC01503, LINC02084, RAB11B-AS1, Z97200.1) based on the data presented by the Cox and LASSO regression analyses conducted for predicting the prognosis, immune response, immunotherapy and chemotherapy response for HNSCC patients. More specifically, AC015878.1 was seen to be a member of the stemness-related model for HNSCC [22], while EMSLR and Z97200.1 were seen to be important components of the prognostic signature used for bladder cancer and kidney renal clear cell carcinoma, respectively [23, 24]. EMSLR regulated the cell proliferation and differentiation by repressing the promoter activity of LncPRESS1 in lung cancer cell [25]. LINC01503 could promoted the proliferation, migration, and invasion in esophageal squamous cell carcinoma (ESCC) cell lines. It disrupted the interaction of EBP1 and the subunit of PI3K, and then increased the AKT signaling [26]. Furthermore, LINC02084 was used as a risk predictor in kidney renal clear cell carcinoma, colon cancer, and hepatocellular carcinoma [27–29]. Moreover, LINC01503 could facilitate cell migration, infiltration, and epithelial–mesenchymal transition in cholangiocarcinoma cells [30], whereas CYTOR was up-regulated and significantly associated with the poor prognosis of the cancer patients[31], In HNSCC, CYTOR inhibited cell apoptosis following treatment with the chemotherapeutic drug diamminedichloroplatinum (DDP) [32]. In addition, RAB11B-AS1 was observed to be important for metastasis and poor prognosis in tumor cells [33, 34]. These studies suggested that the 7 anoikis-related lncRNAs could be advantageous in the construction of prognostic models. Further analysis of these lncRNAs could present novel targets for developing effective strategies for tumor therapy.

This model helped in categorizing the HNSCC patients into both risk groups on the basis of their median risk scores. This prognostic anoikis-related lncRNA signature was seen to be a better discriminator for HNSCC patients compared to the whole genome, anoikis-related genes, and anoikis-related lncRNAs. Therefore, we conducted a comprehensive analysis and evaluation of the proposed risk model for forecasting the prognosis and OS of HNSCC patients. K–M curve analysis showed that low-risk patients showed significantly better OS, DSS, and PFS values than those displayed by high-risk patients. K–M analysis of the clinical subgroup characteristics stated that low-risk patients showed significantly higher OS values. Furthermore, the results of the Cox regression analyses highlighted the fact that the proposed risk model could serve as an independent prognosis-predictive indicator in HNSCC patients using the data acquired from the training, validation, and entire sets. The proposed risk score-based nomogram offered findings that validated its predictive value for HNSCC patients. These results highlighted the effective role played by the anoikis-related lncRNA signature in anticipating the prognosis and OS of patients, suggesting that the proposed risk model complemented the clinicopathological characterization methods.

The Food and Drug Administration (FDA) proposed the application of TMB as a clinical biomarker for determining the ICB response in solid tumors, however, very few studies determined the predictive power of TMB in HNSCC patients [35]. Therefore, we determined the link between TMB and the risk model in HNSCC tissues based on mutation data derived from TCGA. The TMB score was computed after dividing the total sum of somatic mutations by size of exomes, and the findings implied that the high-risk HNSCC patients showed a high number of somatic mutations in comparison to low-risk HNSCC patients, specifically for *TP53*. Loss of *TP53* might influence the survival of tumors after radiation or chemotherapy and it could influence the patient’s prognosis [36]. HNSCC samples were categorized into the high- and low-TMB groups on the basis of their median TMB scores. A high TMB score was significantly related to poor outcomes, but it must be noted that low-risk HNSCC patients exhibited a better prognosis irrespective of their TMB score. This finding further indicated that the proposed risk model could act as an independent candidate for anticipating the prognosis of HNSCC patients.

TME contributes significantly to tumor progression, especially during tumor initiation and metastasis, since the infiltration of the immune cells around the malignant tissues was sensitive to detecting cancer cells and inhibiting their growth [37]. Earlier studies have noted that high immune cell infiltration levels and TME scores were linked to the good prognosis of many cancer patients [38–40]. The TME of HNSCC is distinguished by abnormal changes in immune cell populations, pro-inflammatory cytokines, and immune checkpoint genes [41].

We observed that high-risk HNSCC patients displayed a significantly low immune score in comparison to the low-risk score patients. Furthermore, the low-risk patients exhibited a significantly higher infiltration level of follicular helper T cells, plasma cells, CD8 T cells, resting dendritic cells, regulatory T cells, and resting mast cells, and they also showed an enrichment in the checkpoint, cytolytic activity, HLA, pro-inflammatory, T-cell co-stimulation, and T-cell co-inhibition pathways. In addition, the results of KEGG, GO, and GSEA functional analyses also validated the immune-linked pathways included in the proposed risk model, which enabled us to understand the probable role played by the risk model in anticipating the effect of immunotherapy treatment during clinical studies.

HNSCC is an immunosuppressive disease, however, the development of immunotherapy for HNSCC has progressed rapidly in the past few years [42, 43]. The FDA approved the application of several immune checkpoint inhibitors, such as anti-PD-1 or programmed cell death 1-ligand 1 (PD-L1) antibodies, which include nivolumab and pembrolizumab, durvalumab and atezolizumab for treating the recurrence/metastasis of HNSCC [44]. A few other immune therapies which included the CTLA4 and IDO-1 inhibitors were also evaluated for clinical practice [45]. Though the above treatment strategies showed significant efficacy, very few HNSCC patients benefitted from immunotherapy during clinical practice [46]. Hence, novel prognostic biomarkers need to be identified to determine the immunotherapy response for optimizing the therapeutic strategies. This study noted a significant increase in the IPS values for anti-PD1, anti-CTLA4, and the combined anti-PD1 and anti-CTLA4 immunotherapy in low-risk patients. The expression levels of key immunomodulator or inflammatory mediator ICB genes such as IDO1, CD8A, GZMB, GZMA, PRF1, LAG3, CTLA4, IFNG, and PDCD1 were significantly elevated in low-risk patients. All the findings suggested that low-risk HNSCC patients showed a high sensitivity to the immune checkpoint inhibitors.

Multimodal combination therapies that include radiotherapy, surgery, and chemotherapy can act as the primary treatment strategy for advanced HNSCC patients with poor prognosis owing to recurrence or metastases [45]. Since cisplatin was first introduced in the 1970s, there has been an advancement in the chemotherapeutic strategies for HNSCC patients [47]. Hence, several cytotoxic anti-cancer agents, such as taxane-based anticancer drugs, such as docetaxel and paclitaxel, were more conventionally used for HNSCC [1]. Combined treatment of docetaxel and cisplatin for advanced HNSCC showed a good response of 33–53% [48]. Based on the above data, we employed the pRRophetic algorithm to study the impact of the risk model on the response of four common anti-cancer agents, such as cisplatin, paclitaxel, gemcitabine, and docetaxel. A significantly low IC_50_ value was noted in the high-risk patients for gemcitabine, docetaxel, and cisplatin, which indicated that these patients were more sensitive to chemotherapy. The above findings offered a theoretical basis for formulating personalized treatment regimens for HNSCC. If this finding is validated in a large, multi-centre clinical trial, patients could be accurately stratified based on their risk scores, allowing physicians to tailor treatment strategies and make informed decisions about the use of anti-cancer agents. On the other hand, by identifying those individuals at higher risk of cancer progression or recurrence, physicians would be able to intervene more aggressively with targeted therapies, thereby increasing the chances of successful treatment outcomes.

Although there are some prognostic models for HNSCC, only three anoikis-related models have been reported [9, 49, 50]. Compared with these studies, we constructed the anoikis-related prognostic model from the perspective of lncRNA. Since lncRNA has been proven to have good application in biomarkers for many diseases, the anoikis-related lncRNA signature may be more capable of assessing the prognostic value in HNSCC. However, this study presented a few limitations. First, these sets may not represent the entire HNSCC patient population. Even though we integrated an additional multi-center set, this study remains an in-depth analysis of HNSCC samples from public databases using bioinformatics methods, it is not sufficient for use in clinical practice before further studies and experiments. Second, there could be some bias in the random allocation of samples into the training and validation sets. In addition, the mechanisms of seven prognostic anoikis-related lncRNAs in HNSCC require further investigation.

In conclusion, the risk model that was designed using seven prognostic anoikis-related lncRNAs could anticipate the prognosis of HNSCC patients and can be employed as a good independent predictive indicator for HNSCC patients. Furthermore, this risk model could help in developing immunotherapeutic and chemotherapeutic strategies for treating HNSCC patients. It can help the clinicians develop personalized and precise treatment strategies for HNSCC.

## Data Availability

The data that support the findings of this study are openly available from The Cancer Genome Atlas database (https://portal.gdc.cancer.gov/).

## Abbreviations

HNSCC: Head and neck squamous cell carcinoma
ICB: Immune checkpoint blockade
ECM: Extracellular matrix
lncRNAs: Long non-coding RNAs
PARP: Poly ADP-ribose polymerase
EZH2: Enhancer of zeste homolog 2
TMB: Tumor somatic mutation
TCGA: The Cancer Genome Atlas
LASSO: Least Absolute Shrinkage and Selection Operator
ROC: Receiver operator characteristic
PCA: Principal component analysis
DSS: Disease-specific survival
PFS: Progression-free survival
TME: Tumor microenvironment
ssGSEA: Single sample gene-set enrichment analysis
IPS: Immunophenoscore
TCIA: The Cancer Immunome Atlas
DEGs: Differentially expressed genes
FDR: False discovery rate
GO: Gene ontology
KEGG: Kyoto Encyclopedia of Genes and Genomes
GSEA: Gene Set Enrichment Analysis
HLA: Human leukocyte antigen
PD-1: Programmed cell death-1
CTLA4: Cytotoxic T-lymphocyte associated protein 4
FDA: Food and Drug Administration
OS: Overall survival

## References

[1] Johnson DE, Burtness B, Leemans CR, Lui VWY, Bauman JE, Grandis JR (2020). Head and neck squamous cell carcinoma. Nat Rev Dis Primers.

[2] Chow LQM (2020). Head and neck cancer. N Engl J Med.

[3] Cramer JD, Burtness B, Le QT, Ferris RL (2019). The changing therapeutic landscape of head and neck cancer. Nat Rev Clin Oncol.

[4] Burtness B, Rischin D, Greil R (2022). Pembrolizumab alone or with chemotherapy for recurrent/metastatic head and neck squamous cell carcinoma in KEYNOTE-048: subgroup analysis by programmed death ligand-1 combined positive score. J Clin Oncol.

[5] Cristina V, Herrera-Gómez RG, Szturz P, Espeli V, Siano M (2019). Immunotherapies and future combination strategies for head and neck squamous cell carcinoma. Int J Mol Sci.

[6] Jiang M, Li B (2022). STAT3 and its targeting inhibitors in oral squamous cell carcinoma. Cells.

[7] Frisch SM, Francis H (1994). Disruption of epithelial cell-matrix interactions induces apoptosis. J Cell Biol.

[8] Taddei ML, Giannoni E, Fiaschi T, Chiarugi P (2012). Anoikis: an emerging hallmark in health and diseases. J Pathol.

[9] Qiu L, Tao A, Sun X, Liu F, Ge X, Li C (2023). Comprehensive bioinformatics analysis and experimental validation: an anoikis-related gene prognostic model for targeted drug development in head and neck squamous cell carcinoma. Oncol Res.

[10] Jin L, Chun J, Pan C (2018). The PLAG1-GDH1 axis promotes anoikis resistance and tumor metastasis through CamKK2-AMPK signaling in LKB1-deficient lung cancer. Mol Cell.

[11] Goodall GJ, Wickramasinghe VO (2021). RNA in cancer. Nat Rev Cancer.

[12] Statello L, Guo CJ, Chen LL, Huarte M (2021). Gene regulation by long non-coding RNAs and its biological functions. Nat Rev Mol Cell Biol.

[13] Lu Q, Wang L, Gao Y (2021). lncRNA APOC1P1-3 promoting anoikis-resistance of breast cancer cells. Cancer Cell Int.

[14] Tian H, Lian R, Li Y (2020). AKT-induced lncRNA VAL promotes EMT-independent metastasis through diminishing Trim16-dependent Vimentin degradation. Nat Commun.

[15] Li YR, Fu M, Song YQ, Li SL, Ge XY (2023). Long non-coding RNA MRPL23-AS1 suppresses anoikis in salivary adenoid cystic carcinoma in vitro. Oral Dis.

[16] Dai ZY, Jin SM, Luo HQ, Leng HL, Fang JD (2021). LncRNA HOTAIR regulates anoikis-resistance capacity and spheroid formation of ovarian cancer cells by recruiting EZH2 and influencing H3K27 methylation. Neoplasma.

[17] Chan TA, Yarchoan M, Jaffee E (2019). Development of tumor mutation burden as an immunotherapy biomarker: utility for the oncology clinic. Ann Oncol.

[18] Raeisi M, Zehtabi M, Velaei K, Fayyazpour P, Aghaei N, Mehdizadeh A (2022). Anoikis in cancer: the role of lipid signaling. Cell Biol Int.

[19] Kim H, Choi P, Kim T (2021). Ginsenosides Rk1 and Rg5 inhibit transforming growth factor-β1-induced epithelial-mesenchymal transition and suppress migration, invasion, anoikis resistance, and development of stem-like features in lung cancer. J Ginseng Res.

[20] Dai Y, Zhang X, Ou Y (2023). Anoikis resistance–protagonists of breast cancer cells survive and metastasize after ECM detachment. Cell Commun Signal.

[21] Sattari Fard F, Jalilzadeh N, Mehdizadeh A, Sajjadian F, Velaei K (2023). Understanding and targeting anoikis in metastasis for cancer therapies. Cell Biol Int.

[22] Xu Z, Zhang M, Guo Z (2023). Stemness-related lncRNAs signature as a biologic prognostic model for head and neck squamous cell carcinoma. Apoptosis.

[23] Jiang K, Wu L, Yin X (2022). Prognostic implications of necroptosis-related long noncoding RNA signatures in muscle-invasive bladder cancer. Front Genet.

[24] Zhang X, Qin X, Yu T, Wang K, Chen Y, Xing Q (2022). Chromatin regulators-related lncRNA signature predicting the prognosis of kidney renal clear cell carcinoma and its relationship with immune microenvironment: a study based on bioinformatics and experimental validation. Front Genet.

[25] Priyanka P, Sharma M, Das S, Saxena S (2022). E2F1-induced lncRNA, EMSLR regulates lncRNA LncPRESS1. Sci Rep.

[26] Xie JJ, Jiang YY, Jiang Y (2018). Super-enhancer-driven long non-coding RNA LINC01503, regulated by TP63, is over-expressed and oncogenic in squamous cell carcinoma. Gastroenterology.

[27] Lv Y, Wei W, Huang Z (2018). Long non-coding RNA expression profile can predict early recurrence in hepatocellular carcinoma after curative resection. Hepatol Res.

[28] Lin Y, Xiao Y, Liu S, Hong L, Shao L, Wu J (2022). Role of a lipid metabolism-related lncRNA signature in risk stratification and immune microenvironment for colon cancer. BMC Med Genomics.

[29] Sun Z, Jing C, Xiao C, Li T (2020). Long non-coding RNA profile study identifies an immune-related lncRNA prognostic signature for kidney renal clear cell carcinoma. Front Oncol.

[30] Qu YK, Qu XS, Chen G (2019). LINC01503 promotes cell proliferation, invasion and EMT process in cholangio-carcinoma. Eur Rev Med Pharmacol Sci.

[31] Liang J, Wei X, Liu Z (2018). Long noncoding RNA CYTOR in cancer: a TCGA data review. Clin Chim Acta.

[32] Guo YZ, Sun HH, Wang XT, Wang MT (2018). Transcriptomic analysis reveals key lncRNAs associated with ribosomal biogenesis and epidermis differentiation in head and neck squamous cell carcinoma. J Zhejiang Univ Sci B.

[33] Niu Y, Bao L, Chen Y (2020). HIF2-induced long noncoding RNA RAB11B-AS1 promotes hypoxia-mediated angiogenesis and breast cancer metastasis. Cancer Res.

[34] Chen Z, Liu Z, Yang Y (2018). Long non-coding RNA *RAB11B-AS1* prevents osteosarcoma development and progression via its natural antisense transcript *RAB11B*. Oncotarget.

[35] Valero C, Lee M, Hoen D (2021). Response rates to anti-PD-1 immunotherapy in microsatellite-stable solid tumors with 10 or more mutations per megabase. JAMA Oncol.

[36] Poeta ML, Manola J, Goldwasser MA (2007). TP53 mutations and survival in squamous-cell carcinoma of the head and neck. N Engl J Med.

[37] de Visser KE, Joyce JA (2023). The evolving tumor microenvironment: from cancer initiation to metastatic outgrowth. Cancer Cell.

[38] Kwon JTW, Bryant RJ, Parkes EE (2021). The tumor microenvironment and immune responses in prostate cancer patients. Endocr Relat Cancer.

[39] Tay C, Tanaka A, Sakaguchi S (2023). Tumor-infiltrating regulatory T cells as targets of cancer immunotherapy. Cancer Cell.

[40] Pitt JM, Marabelle A, Eggermont A, Soria JC, Kroemer G, Zitvogel L (2016). Targeting the tumor microenvironment: removing obstruction to anticancer immune responses and immunotherapy. Ann Oncol.

[41] Chen SMY, Krinsky AL, Woolaver RA, Wang X, Chen Z, Wang JH (2020). Tumor immune microenvironment in head and neck cancers. Mol Carcinog.

[42] Yilmaz E, Ismaila N, Bauman JE (2023). Immunotherapy and biomarker testing in recurrent and metastatic head and neck cancers: ASCO Guideline. J Clin Oncol.

[43] Ho AL (2023). Immunotherapy, chemotherapy, or both: options for first-line therapy for patients with recurrent or metastatic head and neck squamous cell carcinoma. J Clin Oncol.

[44] Carlisle JW, Steuer CE, Owonikoko TK, Saba NF (2020). An update on the immune landscape in lung and head and neck cancers. CA Cancer J Clin.

[45] Solomon B, Young RJ, Rischin D (2018). Head and neck squamous cell carcinoma: genomics and emerging biomarkers for immunomodulatory cancer treatments. Semin Cancer Biol.

[46] Runnels J, Bloom JR, Hsieh K (2023). Combining radiotherapy and immunotherapy in head and neck cancer. Biomedicines.

[47] Kish J, Drelichman A, Jacobs J (1982). Clinical trial of cisplatin and 5-FU infusion as initial treatment for advanced squamous cell carcinoma of the head and neck. Cancer Treat Rep.

[48] Patil VM, Noronha V, Menon N (2023). Results of phase III randomized trial for use of docetaxel as a radiosensitizer in patients with head and neck cancer, unsuitable for cisplatin-based chemoradiation. J Clin Oncol.

[49] Wei Z, Zhou C, Shen Y, Deng H, Shen Z (2023). Identification of a new anoikis-related gene signature for prognostic significance in head and neck squamous carcinomas. Medicine (Baltimore).

[50] Chi H, Jiang P, Xu K (2022). A novel anoikis-related gene signature predicts prognosis in patients with head and neck squamous cell carcinoma and reveals immune infiltration. Front Genet.

